# ILC2 activation by keratinocyte-derived IL-25 drives IL-13 production at sites of allergic skin inflammation

**DOI:** 10.1016/j.jaci.2020.02.026

**Published:** 2020-06

**Authors:** Juan Manuel Leyva-Castillo, Claire Galand, Shunya Mashiko, Robert Bissonnette, Alex McGurk, Steven F. Ziegler, Chen Dong, Andrew N.J. McKenzie, Marika Sarfati, Raif S. Geha

**Affiliations:** a Division of Immunology, Boston Children’s Hospital, Harvard Medical School, Boston, Mass; b Immunoregulation Laboratory, Centre de Recherche du Centre Hospitalier de l'Université de Montréal, Montreal, Canada; c Innovaderm Research, Montreal, Québec, Canada; d Immunology Program, Benaroya Research Institute, Seattle, Wash; e Department of Immunology, University of Washington School of Medicine, Seattle, Wash; f Institute for Immunology and School of Medicine, Tsinghua University, Beijing, China; g Beijing Key Lab for Immunological Research on Chronic Diseases, Beijing, China; h Medical Research Council, Laboratory of Molecular Biology, Cambridge, United Kingdom

**Keywords:** Atopic dermatitis, IL-25, IL-13, ILC2, AD, Atopic dermatitis, eGFP, Enhanced green fluorescent protein, IL-25R, IL-25 receptor, ILC, Innate lymphoid cell, ILC2, Type 2 innate lymphoid cell, MC, Mast cell, TEWL, Transepidermal water loss, TSLP, Thymic stromal lymphopoietin, WT, Wild-type

## Abstract

**Background:**

Atopic dermatitis skin lesions demonstrate increased expression of IL-25 by keratinocytes and increased numbers of type 2 innate lymphoid cells (ILC2s) that express high levels of IL-25 receptor (IL-25R). IL-13 is expressed in atopic dermatitis skin lesions and plays an important role in pathogenesis of the disease.

**Objective:**

Our aim was to determine the role of IL-25 and ILC2s in a mouse model of antigen-driven allergic skin inflammation.

**Methods:**

Wild-type mice; mice that express an *Il13-*driven enhanced green fluorescent protein; and mice that lack IL-25R, IL-25 in keratinocytes, or IL-13 or IL-25R in ILC2s were subjected to acute or chronic epicutaneous sensitization with ovalbumin. Sensitized skin was examined by histology for epidermal thickening. Cellular infiltrates were analyzed for surface markers and intracellular expression of enhanced green fluorescent protein by flow cytometry. Gene expression was quantitated by RT quantitative PCR.

**Result:**

In both acute and chronic antigen-driven allergic skin inflammation, signaling by keratinocyte-derived IL-25 in ILC2s is important for epidermal hyperplasia, dermal infiltration by CD4^*+*^ T cells, and cutaneous expression of *Il13* and the IL-13–dependent T_H_2-cell–attracting chemokines *Cc17* and *Ccl22*. ILCs are the major source of IL-13 in acutely sensitized mouse skin, whereas T cells are its major source in chronically sensitized mouse skin.

**Conclusion:**

ILC2 activation by IL-25 is essential for IL-13 expression at sites of allergic skin inflammation.

## Graphical abstract

**Figure:**
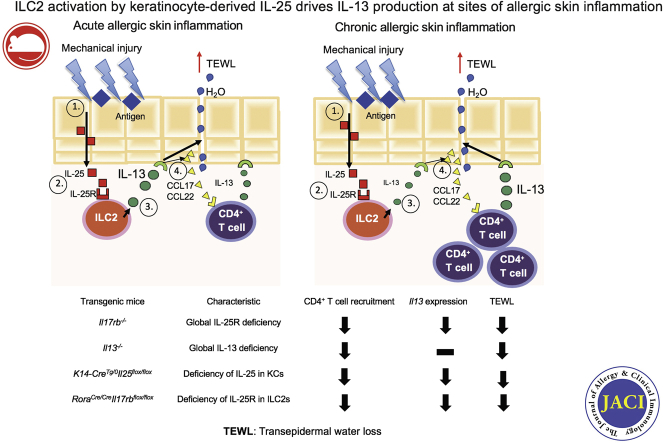

The hallmarks of atopic dermatitis (AD) are defective skin barrier, type-2–dominated cutaneous inflammation, and epidermal hyperplasia.^1^ IL-13 is expressed in AD skin lesions and plays an important role in pathogenesis of the disease.^2^^,^^3^ IL-13 upregulates expression of the T_H_2-cell–attracting chemokines *CCL17* and *CCL22* by human keratinocytes.^4^ IL-13 also suppresses keratinocyte expression of genes important for epidermal integrity, including filaggrin, claudins, and desmoglein-1, which are downregulated in AD skin lesions*.*^5^, ^6^, ^7^, ^8^ Transgenic cutaneous expression of *Il13* drives epidermal hyperplasia, increased expression of T_H_2-cell–attracting chemokines, and recruitment of CD4^+^ T cells.^9^

IL-25 is a member of IL-17 cytokine family produced by epithelial cells, macrophages, eosinophils, mast cells (MCs), and basophils.^10^, ^11^, ^12^ The IL-25 receptor (IL-25R) is a heterodimer of the IL-17RA chain (shared by receptors for other IL-17 family members) and the IL-17 receptor B chain (specific for IL-25R).^10^, ^11^, ^12^ Both nonimmune and immune cells express IL-25R; they include epithelial cells, type 2 innate lymphoid cells (ILC2s), eosinophils, and CD4^+^ T cells.^10^, ^11^, ^12^

Innate lymphoid cells (ILCs) are lymphoid cells that lack lineage markers and antigen-specific surface receptors and are enriched at the interfaces between the body and the environment.^13^ ILC2s express the transcription factor RAR-related orphan receptor alpha, which is essential for their development^14^; the type 2 cytokines IL-5 and IL-13; and receptors for the epithelial-derived cytokines IL-25, thymic stromal lymphopoietin (TSLP), and IL-33.^13^^,^^15^ IL-25, IL-33, and TSLP, alone or in combination, promote IL-13 production by ILC2s *in vitro*.^13^^,^^16^

AD skin lesions demonstrate increased expression of IL-25 by keratinocytes^16^, ^17^, ^18^, ^19^, ^20^ and increased numbers of ILC2s that express high levels of receptors for IL-25, TSLP, and IL-33.^16^^,^^21^ IL-25 alone, or in combination with type 2 cytokines, impairs skin barrier function.^18^^,^^19^^,^^22^^,^^23^ TSLP and IL-33 are important in allergic skin inflammation^24^, ^25^, ^26^; however, the role of IL-25 is not completely understood. We show that ILC2 activation by IL-25 is essential for IL-13 production at sites of allergic skin inflammation.

## Methods

### Mice

*Il17rb*^*–/–*^, *Il13*^*–/–*^, *Il13*^*eGFP/+*^ mice on a BALB/c background were previously described.^27^^,^^28^ *Il17rb*^*fl/fl*^ mice on a BALB/c background were obtained from Dr Ziegler, and *Rora*^*Cre/Cre*^ mice on a C57Bl/6 background were obtained from Dr O’Leary^29^ and crossed on a BALB/c background. IL-25–floxed mice on a C57Bl/6 background were obtained from Dr Dong.^30^ *K14-Cre*^*Tg/0*^ mice on a C57Bl/6 background were obtained from Jackson Laboratories. BALB/c and C57Bl/6 mice were purchased from Charles River Laboratory. All mice were kept in a pathogen-free environment and fed an ovalbumin-free diet. All procedures were performed in accordance with the Animal Care and Use Committee of the Children's Hospital Boston.

### Epicutaneous sensitization

Female mice (aged 6 to 8 weeks) were epicutaneously sensitized for 10 days or 7 weeks, as described previously.^25^^,^^31^ Analyses were done at day 12 or day 49.

### Histology and measurement of epidermal thickness

Skin specimens were fixed in 4% paraformaldehyde embedded in paraffin and analyzed as previously described.^32^

### Mouse skin cell preparation and flow cytometry

**Fig E1:**
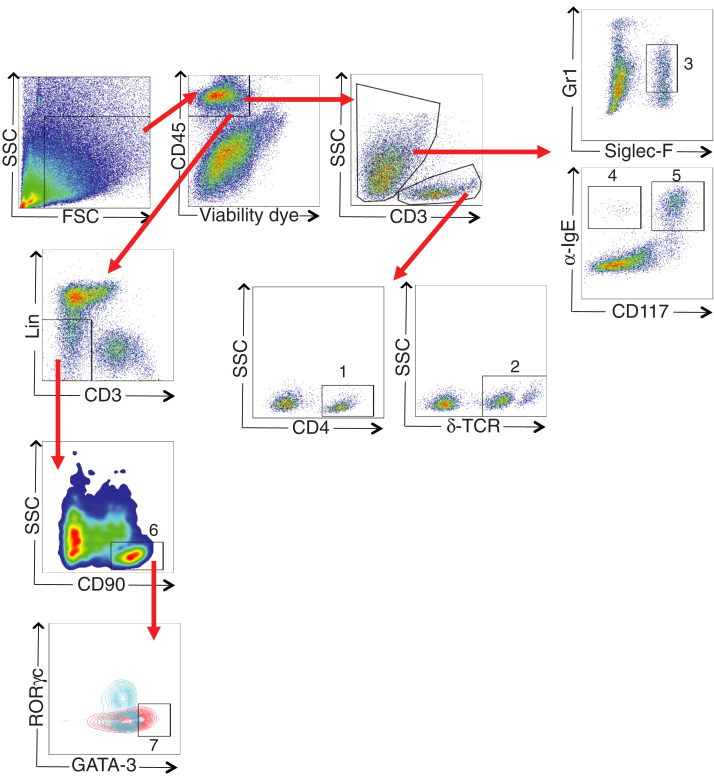
Gating strategy. Representative flow cytometry plots showing gating strategy for CD4^+^ T cells *(1)*, γδ TCR^+^ T cells *(2)*, eosinophils *(3)*, basophils *(4)*, MCs *(5)*, ILCs *(6)*, and ILC2s *(7)*. To gate GATA3^+^ cells in the ILC population (*red*), we used γδ TCR^+^ T cells (*blue*) as a negative control. *FSC,* Forward scatter; *SSC*, side scatter.

Cell isolation from the back skin was performed as previously described.^33^ Cells were preincubated with FcγR-specific blocking mAb (2.4G2) and washed before staining with the following mAbs: B220 (RA3-6B2), CD3 (17A2), CD4 (GK1.5), CD11c (N418), CD19 (1D3), CD45 (30F11), CD90.2 (53-2.1), Gr1 (RB6-8C5), and δ TCR (ebioGL3) from eBioscience (San Diego, Calif); CD11b (M1/70), F4/80 (BM8), and CD117 (2B8) from Biolegend (San Diego, Calif); and anti-IgE (R35-72) from BD Biosciences (San Jose, Calif). BV605 streptavidin from Biolegend was used to detect biotinylated antibodies. Cells were analyzed by flow cytometry by using an LSRFortessa machine (BD Biosciences). The data were analyzed with FlowJo software. CD4^+^ T cells (CD45^+^CD3^+^CD4^+^), γδ TCR^+^ T cells (CD45^+^CD3^+^δTCR^+^), eosinophils (CD45^+^CD3^–^GR1^+^SiglecF^+^), basophils (CD45^+^CD3^–^α–IgE^+^CD117^–^), MCs (CD45^+^CD3^–^α–IgE^+^CD117^+^), ILCs (CD45^+^CD3^–^Lin^–^CD90^+^), and ILC2s (CD45^+^CD3^–^Lin^–^ CD90^+^GATA3^+^) in the skin were identified as shown in Fig E1 (in the Online Repository available at www.jacionline.org).

### mRNA expression analyses

Total skin RNA extraction and measurement of cytokines were performed and analyzed as previously described.^34^

### TEWL

Transepidermal water loss (TEWL) was measured by using a Dermalab instrument DermaLab universal serial bus module (Cortex Technology, Hadsund, Denmark). TEWL was assessed on the epicutaneously sensitized skin, and readings were recorded for 1 minute. The probe was removed from the skin and replaced, after which a second measurement was taken. An average of the 2 readings was used as the TEWL for each mouse.

### Cell culture and *in vitro* cytokine expression

Single-cell suspensions of skin-draining lymph nodes and splenocytes were cultured and stimulated with ovalbumin, and their supernatants analyzed for cytokines by ELISA as previously described.^34^

### Statistical analysis

Statistical significance was determined by a 2-tailed Student *t* test. A *P* value less than .05 was considered statistically significant.

## Results

### IL-25 signaling is required for acute allergic skin inflammation

**Fig 1:**
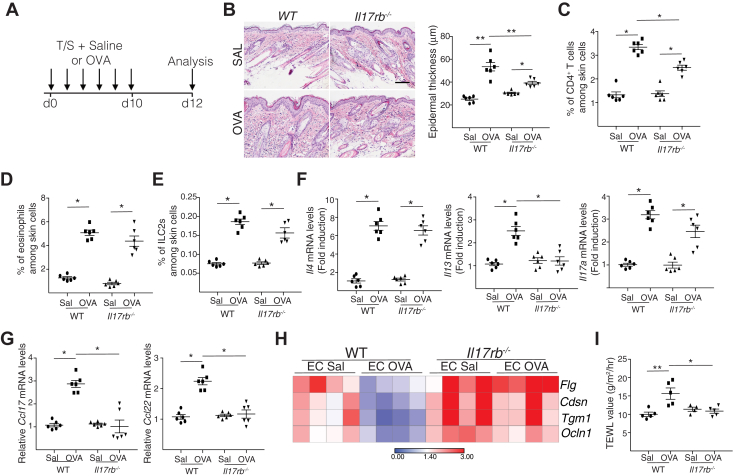
IL-25 signaling is required for acute allergic skin inflammation. **A,** Experimental protocol. **B-I,** Representative hematoxylin and eosin staining (**B** [*left*]) and epidermal thickness measurement (**B** [*right*]); percentages of CD4^+^ T cells (**C**), eosinophils (**D**), and ILC2s (**E**); mRNA levels of *Il4*, *Il13*, *Il17a* (**F**), and *Ccl17* and *Ccl22* (**G**) expressed relative to the mean of saline (*SAL*)-sensitized WT controls; heat map of mRNA levels in epidermal sheets (**H**); and TEWL (**I**) in saline-sensitized and ovalbumin (*OVA*)-sensitized skin of *Il17rb*^*–/–*^ mice and WT controls. Results in **B** to **I** are representative of 2 independent experiments with 4 or 5 mice per group. ∗*P <* .05; ∗∗*P <* .005. *EC*, Epicutaneous; *T/S*, tape stripping.

**Fig E2:**
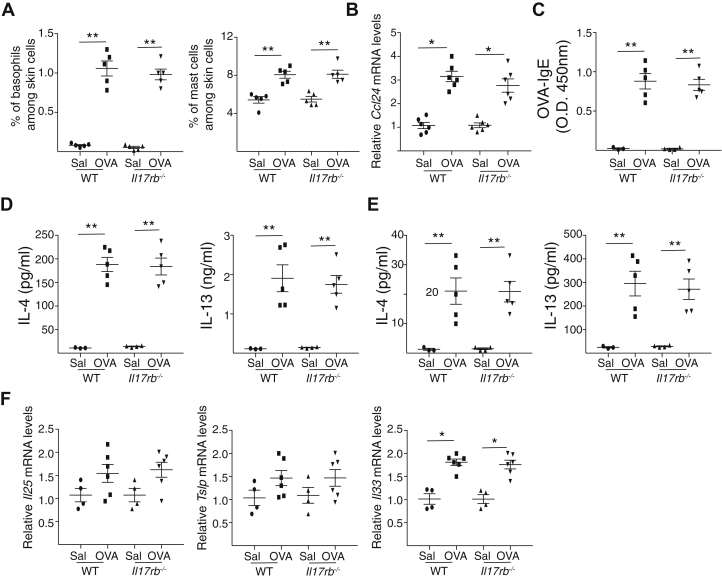
IL-25 signaling is dispensable for accumulation of basophils and MCs at sites of allergic skin inflammation and for local and systemic T_H_2 response induced by epicutaneous sensitization. **A-D,** Percentage of skin basophils and MCs (**A**), serum ovalbumin (*OVA*)-specific IgE levels (**B**), IL-4 and IL-13 secretion by skin-draining lymph nodes (**C**) and splenocytes (**D**) and *Il25*, *Tslp,* and *Il33* mRNA levels in OVA- and saline (*SAL*)-sensitized *Il17rb*^*–/–*^ mice and WT BALB/c controls. Results are representative of 2 independent experiments with 4 or 5 mice per group. *Horizontal lines* represent means ± SEMs. ∗*P <* .05; ∗∗*P <* .005. Calculations performed by using a 2-tailed Student *t* test.

To determine the role of IL-25 in acute allergic skin inflammation, we examined the response of *Il17rb*^*–/–*^ mice to epicutaneous sensitization with ovalbumin over a 12-day period (Fig 1, *A*). *Il17rb*^*–/–*^ mice epicutaneously sensitized with ovalbumin exhibited significantly diminished epidermal thickening and significantly less dermal infiltration by CD4^+^ T cells compared with wild-type (WT) controls (Fig 1, *B* and *C*). Dermal infiltration by eosinophils, basophils, and MCs was comparable in the 2 groups (Fig 1, *D* and see Fig E2, *A* in the Online Repository available at www.jacionline.org). The percentage of CD3^–^Lin^–^CD90^+^GATA3^+^ ILC2s increased to a comparable extent in ovalbumin-sensitized skin of the 2 strains (Fig 1, *E*). These findings demonstrate that IL-25 signaling is important for epidermal thickening and optimal accumulation of CD4^+^ T cells at sites of acute allergic skin inflammation.

Type 2 cytokines are important for epidermal thickening and cutaneous expression by keratinocytes of *Ccl17* and *Ccl22*^4^^,^^9^ (chemokine ligands for CCR4), which is important for the accumulation of T cells in ovalbumin-sensitized mouse skin.^35^ Epicutaneous sensitization of WT BALB/c mice with ovalbumin resulted in local upregulation of *Il4, Il13,* and *Il17a* but not *Ifng* expression (Fig 1, *F* and data not shown). It also resulted in local upregulation of *Ccl17* and *Ccl22* (Fig 1, *G*), as well as *Ccl24,* which encodes eotaxin 2 (see Fig E2, *B*). Upregulation of *Il13, Ccl17,* and *Ccl22* expression was virtually abolished in ovalbumin-sensitized skin of *Il17rb*^*–/–*^ mice compared with in WT controls, whereas upregulation of *Il4, Il17a,* and *Ccl24* expression was unaffected and *Ifng* expression remained unchanged (Fig 1, *F*-*G* and see also Fig E2, *B* and data not shown). These results indicate that IL-25R signaling is essential for *Il13* expression at sites of acute allergic skin inflammation.

The systemic T_H_2 immune response to epicutaneous sensitization with ovalbumin was comparable to that in *Il17rb*^*–/–*^ mice and WT controls. This was evidenced by comparable serum levels of ovalbumin-specific IgE and secretion of IL-4 and IL-13 by skin-draining lymph node cells and splenocytes in response to *in vitro* stimulation with ovalbumin (Fig E2, *C*-*E*). Thus, defective systemic T_H_2 immune response to epicutaneous sensitization cannot explain the failure of *Il17rb*^*–/–*^ mice to upregulate *Il13* expression in the ovalbumin-sensitized skin.

Upregulation of *Il33*, *Il25,* and *Tslp* mRNA expression in the skin following ovalbumin sensitization was comparable in *Il17rb*^*–/–*^ mice and WT controls (Fig E2, *F*). Expression by epidermal sheets of epidermal barrier integrity and the tight junction genes *Flg, Cdsn*, *Tgm1*, and *Ocln1* was downregulated in ovalbumin-sensitized skin compared with in saline-sensitized skin in WT mice but not in *Il17rb*^*–/–*^ mice (Fig 1, *H*). Consistent with this finding, TEWL increased in ovalbumin-sensitized skin compared with in saline-sensitized skin in WT mice but not in *Il17rb*^*–/–*^ mice (Fig 1, *I*). These results demonstrate that IL-25R signaling disrupts epidermal barrier integrity in epicutaneously sensitized skin.

### Keratinocyte-derived IL-25 is required for acute allergic skin inflammation

**Fig E3:**
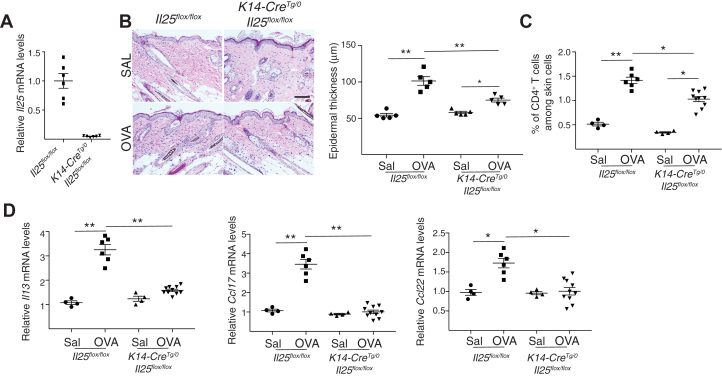
Keratinocyte-derived IL-25 is required for acute allergic skin inflammation. **A.***Il25* mRNA levels in skin from u *K14-Cre*^*Tg/0*^*Il25*^*flox/flox*^ mice and *Il25*^*flox/flox*^ controls expressed relative to the mean of *Il25*^*flox/flox*^ controls. **B-D,** Representative hematoxylin and eosin staining (**B** [*left*]) and epidermal thickness measurement (**B** [*right*]); quantitation of percentage of CD4^+^ T cells (**C**); and mRNA levels of *Il13*, *Ccl17,* and *Ccl22* expressed relative to the mean of saline (*SAL*)-sensitized controls (**D**) in ovalbumin (*OVA*)- and SAL-sensitized skin of *K14-Cre*^*Tg/0*^*Il25*^*flox/flox*^ mice and *Il25*^*flox/flox*^ controls. Results in **A** to **D** are representative of 2 independent experiments with 4 or 5 mice per group. ∗*P <* .05; ∗∗*P <* .005.

We used *K14-Cre*^*Tg/0*^*Il25*^*flox/flox*^ mice, which have selective IL-25 deficiency in keratinocytes, to investigate the role of keratinocyte-derived IL-25 in acute allergic skin inflammation. *Il25* expression in the skin was virtually abolished in *K14-Cre*^*Tg/0*^*Il25*^*flox/flox*^ mice compared with in *Il25*^*flox/flox*^ controls (see Fig E3, *A* in the Online Repository available at www.jacionline.org). Ovalbumin-sensitized skin from *K14-Cre*^*Tg/0*^*Il25*^*flox/flox*^ mice demonstrated significantly reduced epidermal thickening, infiltration by CD4^+^ T cells, and expression of *Il13, Ccl17*, and *Ccl22* mRNA compared with in *Il25*^*flox/flox*^ controls (Fig E3, *B*-*D*). Infiltration with eosinophils and *Il4* expression were comparable in the 2 strains (data not shown). These results implicate keratinocytes as the source of IL-25, which promotes acute allergic skin inflammation.

### The phenotype of acute allergic skin inflammation in IL-13–deficient mice recapitulates that of IL-25R–deficient mice

**Fig 2:**
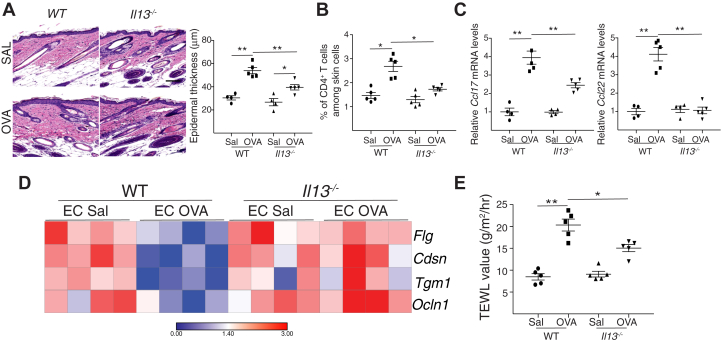
Acute allergic skin inflammation in IL-13–deficient mice recapitulates that in IL-25R–deficient mice. **A-E,** Representative hematoxylin and eosin staining (**A** [*left*]) and epidermal thickness measurement (**A** [*right*]), quantitation of percentage of CD4^+^ T cells (**B**) and mRNA levels of *Ccl17* and *Ccl22* expressed relative to the mean of WT controls, and (**C**) heat map of mRNA levels in epidermal sheets (**D**) and TEWL (**E**) in ovalbumin (*OVA*)- and saline (*SAL*)-sensitized skin of *Il13*^*–/–*^ mice and WT controls Results in **A** to **E** are representative of 2 independent experiments with 4 or 5 mice per group. ∗*P <* .05; ∗∗*P <* .005. *EC*, Epicutaneous.

Epidermal thickening, accumulation of CD4^+^ T cells, and expression of *Ccl17* and *Ccl22* were all significantly diminished in ovalbumin-sensitized skin of *Il13*^*–/–*^ mice compared with in WT controls (Fig 2, *A*-*C*). Importantly, epidermal sheets from ovalbumin-sensitized skin of *Il13*^*–/–*^ mice exhibited increased mRNA levels of *Flg*, *Cdsn*, *Tgm1*, and *Ocln1* compared with the levels in their WT counterparts (Fig 2, *D*, *E*). TEWL in ovalbumin-sensitized skin was significantly lower in *Il13*^*–/–*^ mice than in WT controls (Fig 2, *E*). The similarity of the phenotype of ovalbumin-sensitized skin in *Il13*^*–/–*^ and *Il17rb*^*–/–*^ mice suggests that IL-13 mediates the ability of IL-25 to promote acute allergic skin inflammation.

### ILCs are the major source of IL-13 in acute allergic skin inflammation

**Fig 3:**
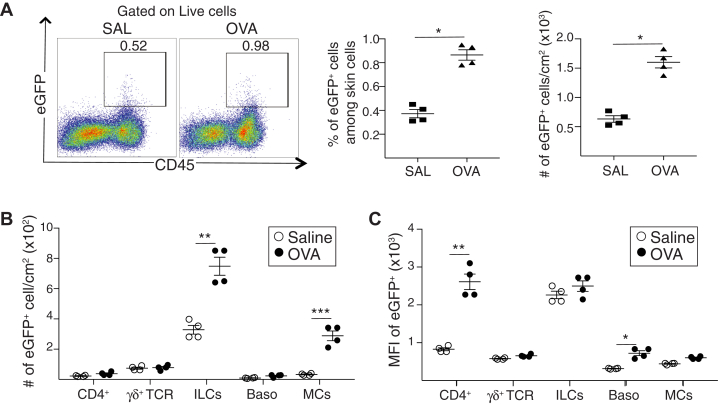
ILCs are the major source of IL-13 in acute allergic skin inflammation. **A-C,** Representative flow cytometry plot (*left*), percentage (*center*), and number (*right*) of eGFP^+^ cells (**A**); numbers of eGFP^+^ cells (**B**); and mean fluorescence intensity (*MFI*) of eGFP expression (**C**) for CD4^+^ T cells, TCRγδ^+^ T cells, ILCs, basophils (*Baso*), and MCs in epicutaneously sensitized skin of *Il13*^*egpf/+*^ mice. Results in **A** to **C** are representative of 2 independent experiments with 4 or 5 mice per group. ∗*P <* .05; ∗∗*P <* .005. *SAL*, Saline; *OVA*, ovalbumin.

**Fig E4:**
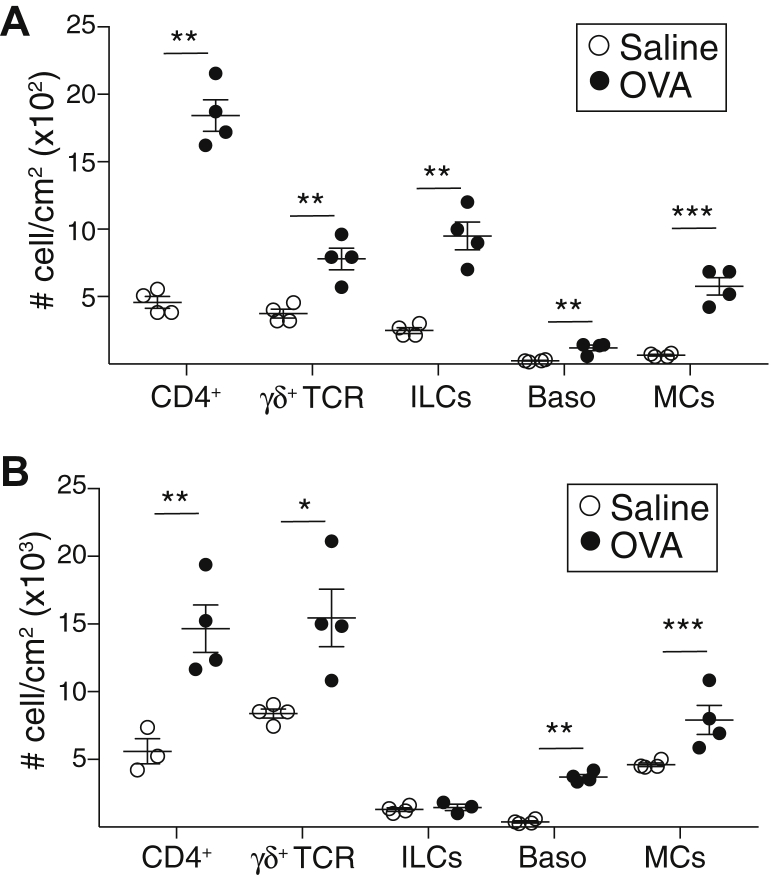
Acute and chronic epicutaneous sensitization with ovalbumin (*OVA*) induced an increase in CD4^+^ T cells, TCRγδ^+^ cells, basophils (*Baso*), and MCs. **A** and **B,** Number of cells for CD4^+^ T cells, TCRγδ^+^ T cells, ILCs, Baso, and MCs in skin of *Il13*^*egpf/+*^ mice following acute (**A**) and chronic (**B**) epicutaneous sensitization. Results are representative of 2 independent experiments with 4 mice per group. *Horizontal lines* represent means ± SEMs. ∗∗*P <* .005; ∗∗∗ *P <* .001. Calculations performed by using a 2-tailed Student *t* test.

To identify the cellular sources of IL-13 in acute allergic skin inflammation, we used *Il13*^*eGFP*^ reporter mice, in which the IL-13 promoter drives the expression of enhanced green fluorescent protein (eGFP).^28^ Flow cytometry analysis revealed that acute epicutaneous sensitization with ovalbumin resulted in a significant increase in the number of CD4^+^ T cells, TCRγδ^+^ cells, ILCs, basophils, and MCs (see Fig E4, *A* in the Online Repository available at www.jacionline.org). In addition, epicutaneous sensitization with ovalbumin caused an approximately 2-fold increase in the percentage and number of CD45^+^eGFP^+^(IL-13^+^) cells in the skin of *Il13*^*eGFP*^ mice compared with in the controls (Fig 3, *A*). ILCs were the most abundant eGFP^+^ cells and had the highest expression of eGFP (IL-13) in saline-sensitized skin (Fig 3, *B* and *C*). CD4^+^ cells, TCRγδ^+^ cells, basophils, and cKIT^+^IgE^+^ MCs each accounted for a small fraction of eGFP^+^(IL-13^+^) cells in saline-sensitized skin and expressed eGFP (IL-13) at levels lower by 3-fold or more compared with those in ILCs (Fig 3, *B* and *C*). Ovalbumin sensitization caused a significant increase in the numbers of eGFP^+^ (IL-13^+^) ILCs and MCs but not in the numbers of eGFP^+^ (IL-13^+^) CD4^+^ T cells, TCRγδ^+^ cells, or basophils (Fig 3, B). It did not alter eGFP (IL-13) expression in ILCs, TCRγδ^+^ cells, or MCs, but it did cause a 3-fold increase in eGFP (IL-13) expression in CD4^+^ T cells that reached the level of eGFP (IL-13) expression in ILCs (Fig 3, *C*). Ovalbumin sensitization slightly increased eGFP (IL-13) expression in basophils (Fig 3, *C*). These results indicate that ILCs are the major IL-13^+^ cell population at sites of acute allergic skin inflammation.

### IL-25 acts directly on ILC2s to drive acute allergic skin inflammation

**Fig 4:**
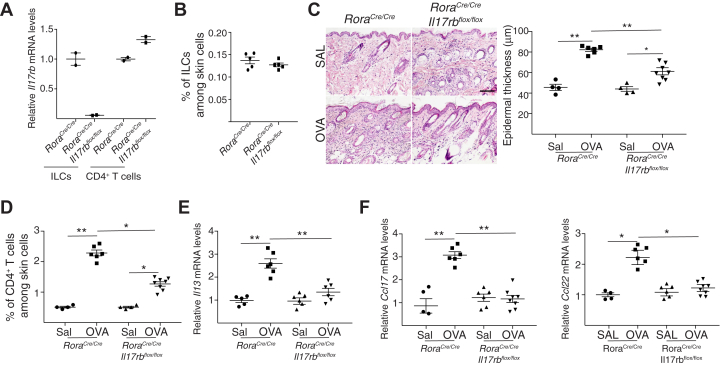
IL-25 acts directly on ILC2s to drive acute allergic skin inflammation. **A** and **B.***Il17rb* mRNA levels in sorted skin ILCs and skin CD4^+^ T cells (**A**) and percentage of skin ILC2s (**B**) in *Rora*^*Cre/Cre*^*Il17rb*^*flox/flox*^ mice and *Rora*^*Cre/Cre*^ controls. **C-F,** Representative hematoxylin and eosin staining (**C** [*left*]) and epidermal thickness measurement (**C** [*right*]); quantitation of percentage of CD4^+^ T cells (**D**); and mRNA levels of *Il13* (**E**), *Ccl17,* and *Ccl22* (**F**) in acutely epicutaneously sensitized skin of *Rora*^*Cre/Cre*^*Il17rb*^*flox/flox*^ mice and *Rora*^*Cre/Cre*^ controls. Values in **D** and **E** are expressed relative to the mean of saline (*SAL*)-sensitized controls. Results in **A** to **F** are representative of 2 independent experiments with 4 or 5 mice per group. ∗ *P <* .05; ∗∗ *P <* .005. *OVA*, Ovalbumin.

To investigate whether IL-25 acts directly on ILC2s to drive acute allergic skin inflammation, we examined *Rora*^*Cre/Cre*^*Il17rb*^*flox/flox*^ mice, which selectively lack IL-25R expression in ILC2s. *Il17rb* mRNA expression was virtually abolished in CD45^+^Lin^–^CD3^–^CD90^+^ ILCs, but it was preserved in CD4^+^ cells sorted from the skin of *Rora*^*Cre/Cre*^*Il17rb*^*flox/flox*^ mice compared with in *Rora*^*Cre/Cre*^ controls (Fig 4, *A*). The percentage of CD45^+^Lin^–^CD3^–^CD90^+^GATA3^+^ ILC2s in the skin was comparable between the 2 strains (Fig. 4, *B*). Ovalbumin-sensitized skin of *Rora*^*Cre/Cre*^*Il17rb*^*flox/flox*^ mice revealed significantly decreased epidermal hyperplasia, infiltration with CD4^+^ T cells, and expression of *Il13*, *Ccl17*, and *Ccl22* mRNA compared with in *Rora*^*Cre/Cre*^ controls (Fig 4, *C*-*F*). Infiltration with eosinophils and *Il4* expression were comparable in the 2 strains (data not shown). These results suggest that IL-25 acts directly on ILC2s at sites of acute allergic skin inflammation to promote their production of IL-13.

### T cells are the major source of IL-13 in chronic allergic skin inflammation in mice

**Fig 5:**
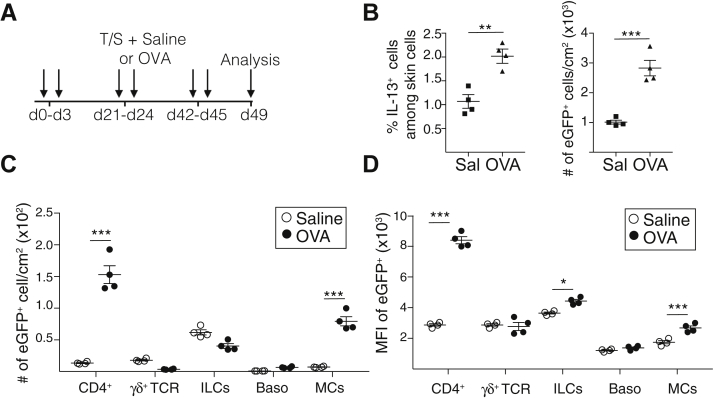
T cells are the major source of IL-13 in chronic allergic skin inflammation in mice. **A,** Experimental protocol for induction of chronic allergic skin inflammation in mice. **B-D,** Percentage (**B** [*left*]) and number (**B** [*right*]) of eGFP^+^ cells; numbers of eGFP^+^ cells (**C**); and mean fluorescence intensity (*MFI*) of eGFP expression (**D**) for CD4^+^ T cells, TCRγδ^+^ T cells, ILCs, basophils (*Baso*), and MCs in the skin of *Il13*^*egpf/+*^ mice subjected to chronic epicutaneous sensitization with ovalbumin (*OVA*). Results in **B**-**D** are representative of 2 independent experiments with 4 or 5 mice per group. *Horizontal lines* in **B**-**D** represent means. ∗*P <* .05; ∗∗*P <* .005; ∗∗∗*P <* .001. *SAL*, Saline.

Adult patients with AD have chronic disease, and T cells are the major source of IL-13 in lesional skin.^36^ This observation prompted us to investigate the cellular source of IL-13 in a mouse model of chronic allergic skin inflammation elicited by repeated application of ovalbumin to tape-stripped skin of *Il13*^*eGFP*^ mice over a 7-week period (Fig 5, *A*). We previously showed that *Il13* expression is significantly increased in ovalbumin-sensitized skin in this model of chronic sensitization.^37^ Flow cytometry analysis revealed that chronic epicutaneous sensitization with ovalbumin resulted in a significant increase in the number of CD4^+^ T cells, TCRγδ^+^ cells, ILCs, basophils, and MCs (Fig E4, *B*). In addition, chronic epicutaneous sensitization with ovalbumin caused a significant (approximately 2-fold) increase in the percentage and number of CD45^+^eGFP^+^(IL-13^+^) cells in the skin (Fig 5, *B*). The majority were CD4^+^ T cells (55%), followed by MCs (28%), ILCs (15%), γδ T cells (<5%), and basophils (<5%) (Fig 5, *C*). CD4^+^ T cells had the highest mean fluorescence intensity of IL-13 expression, followed by ILCs and MCs (Fig 5, *D*). The number of CD4^+^IL-13^+^ cells was 10-fold higher and their IL-13 mean fluorescence intensity of IL-13 expression was 4-fold higher in skin chronically sensitized with ovalbumin than in acutely sensitized skin. These results indicate that T cells are the major source of IL-13 in antigen-driven chronic allergic skin inflammation in mice.

### Cutaneous accumulation of CD4^+^ T cells and *Il13* expression in chronic allergic skin inflammation is dependent on ILC2 activation by IL-25

**Fig 6:**
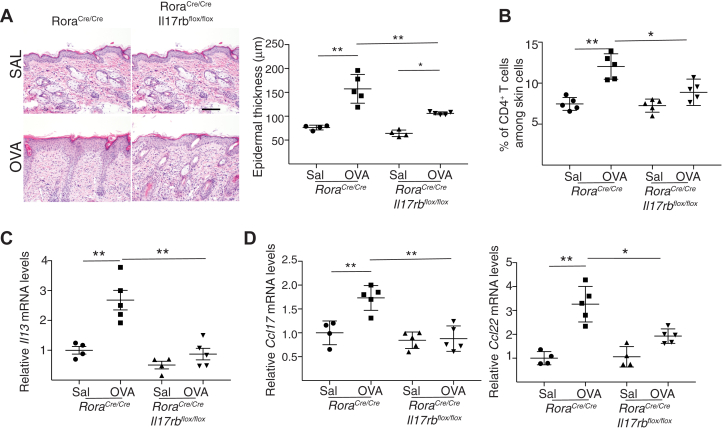
IL-17 receptor B (*IL-17rb*) expression by ILC2s is required to drive chronic allergic skin inflammation. **A**-**D**, Representative hematoxylin and eosin staining (**A** [*left*]) and epidermal thickness measurement (**A** [**right**]); quantitation of percentage of CD4^+^ T cells (**B**); and mRNA levels of *Il13* (**C**), *Ccl17,* and *Ccl22* (**D**) in skin of *Rora*^*Cre/Cre*^*Il17rb*^*flox/flox*^ mice and *Rora*^*Cre/Cre*^ controls mice subjected to chronic epicutaneous sensitization with ovalbumin (*OVA*). Values in **C** and **D** are expressed relative to the mean of saline (SAL)-sensitized controls. Results in **A**-**D** are representative of 2 independent experiments with 4 or 5 mice per group. ∗*P <* .05; ∗∗ *P <* .005. *N.D.*, Not detected.

We asked whether IL25 activation of ILC2s is important for the accumulation of CD4^+^ T cells at sites of chronic allergic skin inflammation. Analysis of the skin of mice subjected to chronic epicutaneous sensitization with ovalbumin revealed that epidermal thickening, skin infiltration by CD4^+^ T cells, and upregulation of expression of *Il13*, *Ccl17,* and *Ccl22* mRNA were significantly reduced or virtually abolished in *Rora*^*Cre/Cre*^*Il17rb*^*flox/flox*^ mice compared with in *Rora*^*Cre/Cre*^ controls (Fig 6, *A*-*D*). Infiltration with eosinophils and *Il4* expression were comparable in the 2 strains (data not shown). Because T cells were by far the major source of IL-13 in skin chronically sensitized with ovalbumin, the lack of upregulation of *Il13* expression in *Rora*^*Cre/Cre*^*Il17rb*^*flox/flox*^ mice suggests that IL-25 activation of ILC2s is necessary for the accumulation of CD4^+^IL-13^+^ T cells at sites of chronic allergic skin inflammation in mice.

## Discussion

We demonstrate that keratinocyte-derived IL-25 activates ILC2s directly to promote IL-13 production, thereby also activating epidermal hyperplasia and CD4^+^ T-cell accumulation in acute as well as chronic antigen-driven allergic skin inflammation.

Our findings in *Il17rb*^*–/–*^ mice demonstrate that IL-25 signaling is essential for the upregulation of *Il13* expression at sites of acute allergic skin inflammation caused by short-term (12 days) epicutaneous sensitization of mouse skin with ovalbumin. Consistent with the decreased expression of *Il13*, IL-13–dependent epidermal hyperplasia and expression of the T-cell–attracting chemokines *Ccl17* and *Ccl22,* as well as the recruitment of CD4^+^ T cells, were all decreased in the ovalbumin-sensitized skin of *Il17rb*^*–/–*^ mice. Expression of the epidermal integrity genes *Flg*, *Cdsn*, *Tgm1,* and *Ocln,* which are known to be downregulated by IL-13, decreased in WT mice but remained unaltered in *Il17rb*^*–/–*^ mice. In parallel, TEWL increased in WT mice but remained unaltered in *Il17rb*^*–/–*^ mice. Ovalbumin-sensitized skin of *Il13*^*–/–*^ mice phenocopied that of *Il17rb*^*–/–*^ mice, strongly suggesting that the changes observed in *Il17rb*^*–/–*^ mice were due to failure to upregulate *Il13* expression in epicutaneously sensitized skin. *Il17rb*^*–/–*^ mice showed a greater reduction in epidermal hyperplasia and TEWL than did *Il13*^*–/–*^ mice, suggesting that factors in addition to IL-13 may mediate the effect of IL-25 in antigen-driven acute allergic skin inflammation. A recent report indicates that IL-25 plays an important role in keratinocytes in a mouse model of psoriatic inflammation mediated by a type 17 immune response.^23^ We have shown that IL-22 and IL-17A play an important role in promoting epidermal hyperplasia induced by epicutaneous sensitization.^32^ We do not exclude the possibility that IL-25 could promote epidermal hyperplasia and skin barrier defects in our model by IL-13–independent mechanisms.

Previous studies *in vitro* indicate that IL-25 plays a role in the generation and maintenance of T_H_2 cells.^10^^,^^38^ Our findings in *Il17rb*^*–/–*^ mice demonstrate that IL-25 signaling is dispensable for generation of the local and systemic T_H_2 response induced by epicutaneous sensitization with ovalbumin. In agreement with our results, IL-25 was found to not be important for *in vivo* T_H_2 polarization induced by intradermal or subcutaneous *Nippostrongylus brasiliensis* infection.^39^ Collectively, these observations suggest that the role of IL-25 in T_H_2 polarization is bypassed by multiple nonredundant mechanisms generated *in vivo* during parasite infection and allergic inflammation.

IL-25 signaling was not important for the expansion of ILC2s, nor was it important for accumulation of eosinophils, basophils, and MCs or upregulation of *Il4* expression in ovalbumin-sensitized skin. ILC2s expand in response to cytokines that, in addition to including IL-25, also include IL-33, TSLP, and IL-2. Normal upregulation of *Il33* and *Tslp* expression may have been sufficient to support expansion of ILC2s in the ovalbumin-sensitized skin of *Il17rb*^*–/–*^ mice. We found that basophils are the major source of *Il4* mRNA in our mouse model of acute AD, as evidenced by RT quantitative PCR analysis of sorted skin cells (unpublished observations), which likely explains the normal upregulation of *Il4* expression and the IL-4 downstream target *Ccl24*^40^ in ovalbumin-sensitized skin of acutely sensitized *Il17rb*^*–/–*^ mice. Normal upregulation of *Il4* and *Ccl24* expression may explain the normal eosinophil skin infiltration in ovalbumin-sensitized skin of *Il17rb*^*–/–*^ mice.

The phenotype of ovalbumin-sensitized skin of mice with selective deficiency of *Il25* in keratinocytes, or selective deficiency of *Il17rb* in ILC2s, recapitulated that in *Il17rb*^*–/–*^ mice, indicating that keratinocyte-derived IL-25 acts directly on ILC2s to upregulate *Il13* production in ovalbumin-sensitized skin. Importantly, we demonstrated that ILCs are the major source of cutaneous IL-13 in mouse acute allergic skin inflammation. Consistent with our results, ILC2s are also the major source of IL-13 in the lungs of mice undergoing acute airway inflammation induced by papain, house dust mite, or ovalbumin.^28^^,^^41^^,^^42^ Skin ILC2s express receptors for TSLP, IL-25, and IL-33, and they produce IL-13 in response to *in vitro* stimulation with these cytokines.^43^ Moreover, lack of ILC2s attenuates the skin inflammation elicited in mice by transgenic expression IL-33 in keratinocytes,^44^ or by application of MC903,^16^^,^^21^ which promotes keratinocyte-derived TSLP overproduction.^25^ Our results showed that *Il13* expression is virtually abolished in the acutely epicutaneously sensitized skin of *Il17rb*^*–/–*^ mice despite normal upregulation of *Tslp* and *Il33* expression, suggesting that IL-25 plays a nonredundant role in driving *Il13* expression by skin ILC2s.

T cells, rather than ILC2s, were the major source of IL-13 in the skin of mice epicutaneously sensitized with ovalbumin chronically for 7 weeks. Nevertheless, IL-25 activation of ILC2s was critical for the accumulation of CD4^+^ cells and upregulation of *Il13* expression, and thereby for epidermal hyperplasia in chronically sensitized mouse skin, because all of these events were reduced or abolished in *Rora*^*Cre/Cre*^*Il17rb*^*flox/flox*^ mice. In addition to its expression by ILC2s, *Rora* is expressed by a subpopulation of T_H_2 cells and regulatory T cells.^45^^,^^46^ However, the systemic T_H_2 response to epicutaneous sensitization was unaffected in *Il17rb*^*–/–*^ mice with global IL-25R deficiency. Thus, IL-25R deficiency in cells other than ILC2s is unlikely to explain the defect in *Il13* expression at sites of chronic EC sensitization in *Rora*^*Cre/Cre*^*Il17rb*^*flox/flox*^ mice. Rather, the results suggest that IL-25 activation of ILC2s plays an important role in chronic allergic skin inflammation.

Our results provide evidence for a crosstalk in allergic skin inflammation between keratinocytes producing IL-25 and ILC2s to promote the production of IL-13, which in turn acts back on keratinocytes to promote their proliferation and production of T-cell–attracting chemokines. A similar ILC2-epithelial cell crosstalk has been demonstrated during helminthic infection in the small intestine.^47^, ^48^, ^49^ Collectively, these observations support a paradigm in which a crosstalk between ILC2s and epithelial cells promotes immunity at mucosal barriers.

**Clinical implications**

ILC2 activation by IL-25 drives cutaneous IL-13 production in allergic skin inflammation. IL-25 blockade may be beneficial in patients with AD.
