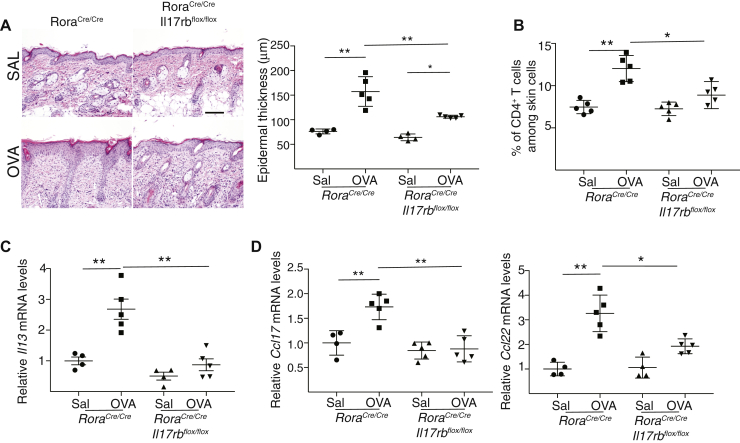# Corrigendum

**DOI:** 10.1016/j.jaci.2022.01.015

**Published:** 2022-03

With regard to the article in the June 2020 issue entitled “ILC2 activation by keratinocyte-derived IL-25 drives IL-13 production at sites of allergic skin inflammation” (J Allergy Clin Immunol 2020;145:1606-14.e4) the authors report an error in Fig 6, *A*. The authors regret that Fig 6, *A* of this paper has a duplication error in the photomicrographs of saline-sensitized skin from *Rora*^*cre/cre*^*/Il17rb*^*flox/flox*^ mice and *Rora*^*c*re/cre^ controls*.* The corrected Fig 6 is below. This did not impact the analysis, results, or conclusions of the article. The authors regret this error and apologize for any inconvenience.

**Figure:**